# A rare coexistence of concurrent breast hemangioma with fibroadenoma: a case report

**DOI:** 10.1186/1757-1626-2-7005

**Published:** 2009-05-15

**Authors:** Vibha Kawatra, Akhila Lakshmikantha, Kajal Kiran Dhingra, Parul Gupta, Nita Khurana

**Affiliations:** Department of Pathology, Maulana Azad Medical College and Lok Nayak HospitalBahadur Shah Zafar Marg, New Delhi -110002India

## Abstract

We report the case of a 38-year-old Asian, Indian female with capillary hemangioma breast in coexistence with the commonly occurring fibroadenoma. Clinical examination of the breast revealed a 4 cm diameter lump. Mammography revealed a well defined slightly hypoechoic lesion with smooth contours. A lumpectomy was performed. Histopathology confirmed the diagnosis of a completely encapsulated fibroadenoma coexistent with a capillary hemangioma in the adjacent breast tissue. The rarity of literature on breast hemangioma especially capillary type with coexisting fibroadenoma deserves mention.

## Introduction

Vascular tumors of the breast are uncommon and include angiosarcomas and hemangiomas of which angiosarcomas are more common. Hemangiomas are benign vascular tumors that are rarely seen in the breast although they have been found incidentally on microscopy of biopsy material for other indications [1,2]. We present a case of 38-year-old female with a breast lump of 3 months duration that was clinically and cytologically suspected to be a fibroadenoma but incidentally showed a coexistent capillary hemangioma on lumpectomy.

## Case presentation

A 38-year old female Asian, Indian patient presented to the surgical out patient department with complaints of a right sided breast lump for three months. There was no history of prior breast mass, pain, trauma, bleeding, discharge, hormone intake or family history of breast cancer. On examination, there was a single well-defined 4 cm diameter, non-tender, firm, mobile mass in the right upper outer quadrant. There was no retraction or ulceration of the overlying skin. Ultrasonography revealed a round, slightly hypoechoic lesion with smooth borders, homogeneous internal echoes, absent acoustic shadowing, and normal surrounding tissue, features consistent with fibroadenoma. Mammography showed a well-circumscribed, smooth bordered mass, suggestive of fibroadenoma. Fine needle aspiration yielded hemorrhagic aspirate with benign ductal cells and stromal fragments suggestive of a fibroadenoma. Even though the patient was assured by the physician of the clinical and cytological diagnosis favoring benignity of her breast lesion, the patient chose to undergo an urgent removal of the breast mass without going through any further investigative procedures.

**Figure 1.**
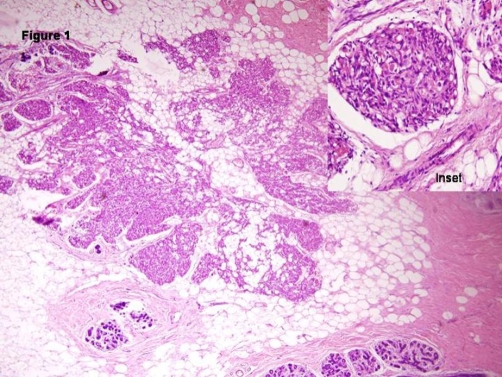
Low power view showing a well circumscribed lobular lesion surrounded by normal breast tissue. (H&EX10x) composed of capillary sized channels, few of them showing red cells. **(Inset)** (H&EX40x).

**Figure 2.**
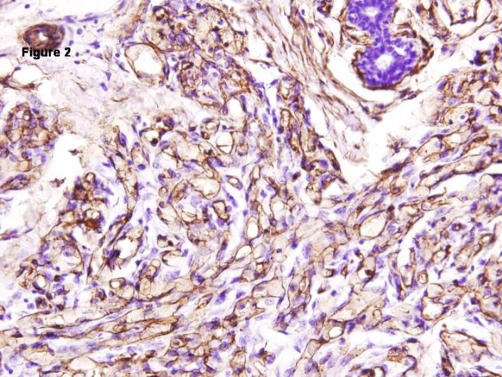
CD34 immunostaining revealing strong reactivity in the lining endothelial cells, confirming the tumor's vascular origin. (H&EX40x).

Grossly we received a fully encapsulated globular mass measuring 4 × 3 × 3 cm with adherent breast tissue. The cut sections through it were firm and revealed slit like areas. In the surrounding breast tissue, we noted few areas of red brown congestion. Sections were taken from both the firm globular mass and the adjacent congested breast tissue. Microscopically, Hematoxylin and Eosin stained sections revealed an intracanalicular type of fibroadenoma with adjacent breast parenchyma revealing small capillary sized blood filled vascular channels with no cytological atypia (Figure 1). A diagnosis of fibroadenoma with co-existent capillary hemangioma was made. A CD 34 immunostain was done to confirm the diagnosis and the endothelial cells lining these vascular channels were found positive for the antibody, thus confirming our diagnosis (Figure 2). On follow up there has been no local recurrence after 1 year of diagnosis.

## Discussion

A hemangioma is a benign vascular tumor usually under 2 cm in size that may be detected by palpation or mammography. They can occur in patients ranging in age from 18 months to 82 years [3]. On mammography the findings of a breast hemangioma are nonspecific and include a normal mammogram or a well-circumscribed hypoechoic or hyperechoic mass with or without calcifications [1,4]. Fine Needle Aspiration cytology is inconclusive in most cases and complete excision and histopathological examination is generally required for diagnosis.

Grossly it is well circumscribed however microscopically it may appear to merge with surrounding tissue, although it never invades or destroys lobules [3,5]. Histologically, there are two common types: the capillary hemangioma composed of proliferating capillary-sized blood vessels and the cavernous hemangioma having large cavernous vascular channels; of which cavernous hemangiomas are more common [6,7]. Hemangiomas are subdivided into 4 types; the perilobular type, parenchymal type, nonparenchymal or subcutaneous, and venous [8,9]. Perilobular hemangioma, a relatively common lesion is generally small and not palpable and occurs in the extralobular stroma in the form of microscopic lesions. Parenchymal hemangiomas are microscopically composed of dilated channels filled with red blood cells that may be divided into lobes by internal fibrous septa, with individual vessels varying in size from capillary to cavernous. Venous hemangiomas are composed largely of venous channels with disorderly vascular proliferation and thick smooth muscle walls [8,9,10]. Nonparenchymal or subcutaneous hemangiomas are located superficial to the anterior pectoral fascia in the subcutaneous fat. Although mammary subcutaneous hemangiomas are benign and are not prone to recurrence or progression to angiosarcoma, complete excision is recommended to exclude the possibility of an underlying angiosarcoma [11]. Atypical hemangiomas are benign breast lesions having histological features as above but with broadly anatomizing vascular channels, endothelial hyperplasia, and/or cytological atypia.

Dener et al [1] reported 2 cases involving the development of parenchymal hemangiomas after exogenous estrogen use. Another reported case involved the formation of an enlarging subcutaneous hemangioma following hormone replacement therapy (HRT) that showed partial involution after discontinuation of HRT [12]. These findings suggest a possible role of estrogen in the development of hemangiomas.

However, in our present case, the patient did not have a history of hormone use. As such, we could not clearly identify a relationship between estrogen intake and the development of the hemangioma similar to previous report of Flis C et al [13].

Other vascular lesions of the breast include angiomatosis, angiosarcoma, post-irradiation atypical vascular lesion. Angiomatosis is a diffuse vascular lesion lacking circumscription. Malignant transformation of hemangioma is rare, but possible. A patient with a history of prior radiotherapy and atypical vascular proliferation is likely to have Post-irradiation atypical vascular lesion. A hemangioma in the breast can histologically be confused with Pseudoangiomatous Stromal Hyperplasia (PASH); however, it is not lined by true lining endothelial cells and will not reveal intraluminal red cells. Moreover CD 34 positivity in our case goes against a diagnosis of PASH.

Stroma of a fibroadenoma like the normal mammary stroma contains connective tissue with a component of resident populations of CD34 positive dendritic interstitial cells and scattered factor XIII positive dendrophages. Some fibroadenomas and phyllodes tumors are known to contain CD34 positive fibroblasts with variable myxoid, collagenous or myofibroblastic differentiation. Exuberant proliferation of these should not be confused with a vascular lesion.

In the management of hemangiomas, imaging follow up is sufficient as these are not likely precursors of angiosarcoma, however a complete excision is recommended to exclude the possibility of an underlying malignant lesion [14,15].

In most of the reported cases, mammography was helpful in diagnosing hemangiomas because of the fine or coarse calcifications they contain, but our patient had no calcifications.

- We conclude that the role of the single diagnostic examination is limited and complementary of all available techniques is required in the evaluation of breast hemangiomas and differentiating them from the more common malignant counterpart, the angiosarcomas.
- Since reports of breast hemangiomas are not common, and most of which are of cavernous type that have been incidentally discovered after biopsy, a thorough examination of the breast tissue should be done for the likelihood of this very rare breast lesion.
